# Editorial: Application of minimally invasive techniques in gastrointestinal diseases in children

**DOI:** 10.3389/fped.2026.1924313

**Published:** 2026-07-23

**Authors:** Fei Liu, Yakun Liu, Jiayu Yan

**Affiliations:** 1 Department of Pediatric Surgery, Guangzhou Institute of Pediatrics, Guangzhou Women and Children’s Medical Center, Guangzhou Medical University, Guangzhou, China; 2 Department of Surgery, Children’s Hospital of Soochow University, Suzhou, Jiangsu, China; 3 Department of General Surgery, Beijing Children’s Hospital, Capital Medical University, National Center for Children’s Health, Beijing, China

**Keywords:** children, endoscopy, gastrointestinal diseases, interventional techniques, laparoscopy, minimally invasive surgery (includes port access), robotic

## Introduction

Over the past two decades, the progressive integration of minimally invasive surgery (MIS) in the management of pediatric gastrointestinal (GI) diseases has represented one of the most significant advancements in pediatric surgical practice (1). MIS transcends being merely a surgical technique and is now a surgical philosophy fundamentally embedded in all facets of pediatric GI disease management (2).

This special issue of *Frontiers in Pediatrics* aimed to explore the value of minimally invasive techniques in treating pediatric GI conditions. The sixteen studies included in this issue evaluate the feasibility, safety, and efficacy of MIS across a broad spectrum of pediatric GI disorders, not only prevalent emergency conditions such as appendicitis and intestinal obstruction, but also complex hepatobiliary and neonatal pathologies, thereby providing a comprehensive snapshot of current practice and underscoring the critical importance of technical refinement, institutional experience, and individualized decision-making.

Biliary atresia and choledochal cysts are relatively common pediatric GI disorders that require advanced surgical expertise (3). The Kasai procedure remains the standard treatment for biliary atresia, requiring precise exposure and meticulous resection of the fibrous portal plate adjacent to the liver hilum (4). Traditionally, the open Kasai approach has been predominant. However, owing to the extensive incisions associated with open surgery and the enhanced visualization afforded by MIS, some pediatric surgeons with substantial MIS experience have begun to perform laparoscopic or robotic Kasai procedures (5). Regarding surgical outcomes, open Kasai has certain advantages, particularly in terms of the time required for fibrous portal plate resection. Conversely, MIS has been shown to significantly reduce the time to initiation of oral feeding and the overall length of hospital stay (Jiang et al.). With increasing surgical experience, patients with biliary atresia undergoing MIS have achieved satisfactory jaundice clearance and native liver survival rates comparable to those treated with open surgery. The application of MIS in the management of choledochal cysts is more widespread. Notably, MIS appears to be applicable across all pediatric age groups for the treatment of choledochal cysts. To achieve the best cosmetic effect, scarless techniques such as single-port laparoscopy are anticipated to gain popularity in the future (Yan et al.). A critical challenge in managing choledochal cysts lies in achieving early diagnosis and timely MIS intervention before the onset of severe complications (6). Collectively, these findings support the early adoption of MIS in selected hepatobiliary cases, provided that surgical teams have overcome the associated learning curves (7).

Appendicitis and intestinal obstruction represent the most common acute abdominal conditions encountered in pediatric populations. MIS has replaced open surgery as the preferred surgical approach for treating various types of appendicitis, and studies have indicated that transumbilical laparoscopic appendectomy can significantly reduce hospital length of stay compared to the traditional three-port laparoscopic surgery (8, 9). In pediatric patients with appendicitis complicated by special comorbidities such as acute leukemia or immunosuppression, a multidisciplinary approach remains advisable: patients with uncomplicated appendicitis may be managed conservatively, while patients with perforated or fecalith-impacted appendicitis warrant prompt intervention via MIS (Liu et al.). For pediatric intestinal obstruction, laparoscopic surgery can also be attempted as the first-line treatment option when the patient's vital signs are stable, facilitating both diagnostic exploration and, when feasible, immediate therapeutic intervention (10). These robust pieces of evidence support MIS as the primary first-line modality for most acute abdominal conditions in children, while emphasizing the necessity for tailored approaches in complex or high-risk cohorts.

Endoscopic techniques, along with laparoscopic and robotic technologies, constitute integral components of MIS and are pivotal in the diagnosis and management of pediatric GI disorders. A narrative review by Zhang et al., featured in this issue, has provided a comprehensive overview of the use of endoscopic techniques in pediatric GI disorders, covering gastroscopy, colonoscopy, and advanced diagnostic and therapeutic modalities, including endoscopic retrograde cholangiopancreatography (ERCP), endoscopic ultrasound (EUS), and peroral endoscopic myotomy (POEM). The study by Qin et al. further corroborates the finding that, despite the technical complexity of ERCP, the procedure achieves a 90.5% success rate in treating pediatric pancreatobiliary disorders. Moreover, endoscopic techniques are considered optimal for managing intraluminal lesions of hollow viscera, including colonic polyps and refractory gastroesophageal reflux disease (11), Povilavičius et al.). Although an increasing number of studies have reported the use of endoscopic techniques to treat conditions like gallstones and appendicitis, the long-term outcomes of these approaches remain uncertain and warrant further longitudinal investigation (12, 13).

Ultrasound-guided interventional puncture is an additional minimally invasive modality used to treat certain rare pediatric GI diseases; however, its use is highly individualized and requires thorough preoperative assessment (6, 14). This research topic confirms that MIS techniques—including laparoscopy, robotic surgery, endoscopy, and interventional procedures—have become indispensable in pediatric GI surgical practice. Nevertheless, their optimal implementation requires rigorous evaluation, ongoing technical advancement, and adherence to a patient-centered, individualized care paradigm. The evidence presented herein is anticipated to inform clinical decision-making, stimulate further research, and ultimately enhance long-term outcomes for children with GI diseases worldwide.
